# Clomiphene citrate versus testosterone replacement therapy in male hypogonadism: a systematic review of literature and meta-analysis

**DOI:** 10.1007/s00228-026-04134-3

**Published:** 2026-07-11

**Authors:** Beatriz T. Constantinou, Bianca C. Benedicto, Lilian Galligani, Beatriz N. Schiavon, Caio M.O Mendes, Maria Micaela G. Petri, Beatriz Uzueli, Ronaldo Soares Maia, Rodrigo Afonso Silva da Sardenberg, Renato Fraietta, Jose Arnaldo Shiomi da Cruz

**Affiliations:** 1 Department of Urology, Ninth of July University (UNINOVE), Sao Bernardo do Campo, Brazil; 2 Department of Urology, Federal University of São Paulo School of Medicine, São Paulo, Brazil; 3 Hapvida NotreDame Research Center, Sao Paulo, Brazil

**Keywords:** Clomiphene, Hormone Replacement Therapy, Testosterone, Hypogonadism, Meta-Analysis

## Abstract

**Introduction:**

Male hypogonadism, including age-related and functional forms, is characterized by insufficient testosterone production often associated with obesity and metabolic comorbidities. Testosterone replacement therapy (TRT) increases serum testosterone but suppresses spermatogenesis and may cause adverse effects. Selective estrogen receptor modulators (SERMs), such as clomiphene citrate, have emerged as an alternative approach to restore endogenous testosterone while preserving fertility. This study aimed to compare the efficacy of clomiphene citrate versus testosterone replacement therapy (TRT) in increasing serum testosterone levels in men with hypogonadism.

**Methods:**

We conducted a systematic review of the literature in PubMed, Embase, Scopus, The Cochrane Library, and Google Scholar to identify studies comparing clomiphene citrate and testosterone replacement therapy (TRT) in men with male hypogonadism’. The primary outcome was the change in serum testosterone levels before and after treatment. Secondary outcomes included variations in other hormonal parameters, and clinical symptom improvement assessed through standardized instruments.

**Results:**

A total of 11 studies with 1512 patients were included (764 on clomiphene citrate; 748 on testosterone replacement therapy, TRT). For serum testosterone, the overall pooled effect showed no significant difference between clomiphene and TRT (MD = 6.64 ng/dL; 95% CI -35.35 to 48.63; I² = 80.6%). Subgroup analyses indicated injectable testosterone achieved higher levels than clomiphene (1 study, 62 patients; MD = -290.50; 95% CI -518.78 to -62.22), whereas gel-based TRT showed no difference (8 studies, 1012 patients; MD = 24.29; 95% CI -18.91 to 67.48; I² = 76.1%). For sexual function, three studies (199 patients; 85 on clomiphene; 114 on TRT) assessing post-treatment libido (ADAM 1–5) showed lower libido scores with clomiphene than TRT (MD = − 0.54; 95% CI − 0.87 to − 0.21; P = 0.009; I² = 18.8%).

**Conclusions:**

No statistically significant difference in serum testosterone levels was observed between clomiphene citrate and testosterone gel, although the evidence is limited by high heterogeneity and some concerns to high risk of bias. TRT was associated with greater improvements in libido. These findings should be interpreted as preliminary, and large-scale randomized trials incorporating clinical and fertility endpoints are needed.

**Supplementary Information:**

The online version contains supplementary material available at 10.1007/s00228-026-04134-3.

## Introduction

Late-onset hypogonadism (LOH) is defined as a clinical and biochemical syndrome characterized by progressive age-related declines in testosterone levels. This condition is more prevalent among specific populations, including individuals with obesity, type 2 diabetes (T2DM), metabolic syndrome (MetS), cardiovascular disease (CVD), chronic obstructive pulmonary disease (COPD), chronic kidney disease, and cancer. The estimated incidence is approximately 11–12 cases per 1,000 men per year [1–4].

According to the American Urological Association (AUA), testosterone deficiency should be considered when total serum testosterone levels fall below 300 ng/dL, with confirmation required on two separate morning samples, due to the circadian cycle of testosterone, processed by the same laboratory and analytical method [5].

Similarly, the European Association of Urology (EAU) recommends that the diagnosis of LOH be based on compatible clinical symptoms and signs accompanied by persistently low testosterone levels. The EAU considers 12 nmol/L (≈ 350 ng/dL) a reliable diagnostic threshold and emphasizes that screening should be limited to symptomatic men [1].

Testosterone replacement therapy (TRT) remains the first-line treatment for male hypogonadism. Although effective in restoring serum testosterone levels, TRT suppresses the hypothalamic–pituitary–gonadal (HPG) axis, resulting in impaired spermatogenesis and potential permanent infertility. In addition, chronic suppression of gonadotropins may result in testicular hypotrophy or atrophy due to reduced intratesticular testosterone production. TRT may also be associated with important adverse effects, including elevated hematocrit, increased serum estrogen, rising prostate-specific antigen (PSA) levels, and changes in lipid profile [6].

To overcome these limitations, selective estrogen receptor modulators (SERMs), have been proposed as alternatives to restore testosterone levels while preserving fertility in men with functional secondary hypogonadism. Among these agents, clomiphene citrate acts by preventing estrogen-mediated negative feedback on the HPG axis, making them particularly beneficial for men with obesity, metabolic disorders and reproductive desire [7, 8].

In recent years, the use of SERMs for male hypogonadism has increased. However, evidence remains limited, and randomized clinical trials show conflicting results regarding their effectiveness in raising testosterone levels and alleviating clinical symptoms. To address this knowledge gap, we conducted a systematic review and meta-analysis to evaluate changes in testosterone levels and hormonal parameters after treatment with clomiphene citrate compared with traditional testosterone replacement therapy in men with hypogonadism.

## Methods

### Search strategy

This systematic review and meta-analysis were performed and reported in accordance with the Cochrane Collaboration Handbook for Systematic Review of interventions and the Preferred reporting Items for Systematic Reviews and Meta-Analysis (PRISMA) Statement guidelines.

We searched PubMed/MEDLINE, Embase, Scopus, the Cochrane Central Register of Controlled Trials (CENTRAL), and Google Scholar from database inception through August 2025 for comparative studies of clomiphene versus testosterone. The search strategy was adapted for each database. Google Scholar was specifically queried to identify grey literature, including conference abstracts and unpublished reports. No language restriction was applied. The core PubMed search strategy was: (“Clomiphene“[Mesh] OR clomiphene OR “clomiphene citrate” OR enclomiphene OR zuclomiphene) AND (“Hypogonadism“[Mesh] OR hypogonadism OR “male hypogonadism” OR “testosterone deficiency” OR “late-onset hypogonadism”) AND (“Testosterone“[Mesh] OR testosterone OR “serum testosterone” OR “total testosterone” OR “free testosterone”).

The references from all included studies, previous systematic reviews and meta-analyses were also searched manually for any additional studies. The prospective meta-analysis protocol was registered on PROSPERO under protocol CRD420251252199.

## Eligibility criteria for study selection

### Inclusion criteria

(1) Randomized trials (RCTs) or non-randomized cohorts (non-RCTs); (2) comparing Clomiphene and Testosterone; (3) adult men with late-onset hypogonadism or testosterone deficiency with baseline total testosterone (TT) levels ≤ 300 ng/dL.

In addition, only studies that reported at least one of the clinical outcomes of interest were included.

### Exclusion criteria

(1) Men with primary hypogonadism; (2) reviews; (3) case reports; (4) opinions, editorials and letters to the editor.

## Endpoints and subgroup analysis

The primary endpoint is the variation in serum testosterone levels (total or free), comparing pre- and post-treatment. Secondarily we analyzed libido clinical symptoms measured by ADAM questionnaire libido domain [9].

## Screening

After deduplication, we used (Clarivate, Philadelphia, PA, USA) and two independent researchers (BC and AS) screened the studies by title and abstract, and disagreements were solved by a third researcher (JA). Following this process, full text screening was performed.

## Data extraction and quality assessment

Two authors (BC and AS) independently extracted the data based on a predefined protocol and disagreements were solved by a third (JA). Risk of bias was assessed in randomized studies using version 2 of the Cochrane Risk of Bias assessment tool (RoB 2). Six non-randomized studies were assessed with the Risk of Bias in Non-randomized Studies – of Interventions tool (ROBINS-I). In 10 studies, two independent authors (BC and BB) performed the risk of bias assessment. Disagreements were resolved by consensus after discussion of the reasons for the discrepancy. Publication bias was investigated using funnel plot analysis of point estimates in relation to study weights.

The certainty of evidence was assessed using the GRADE (Grading of Recommendations Assessment, Development and Evaluation) framework. For each outcome, the certainty rating started at high for randomized controlled trials and low for non-randomized studies. The rating was then downgraded based on five domains: risk of bias, inconsistency, indirectness, imprecision, and publication bias.

### Statistical analysis

Continuous outcomes are presented as mean difference (MD) with a 95% confidence interval (CI). Pooled estimates were calculated using a random-effects model, considering that patients came from different populations. Prediction intervals were included in the forest plots to represent the range within which the true effect of an individual study may lie, given the estimated between-study heterogeneity. Finally, I² heterogeneity, when moderate to high, was addressed through a leave one out analysis and a Baujat plot.

Outcomes with at least ten studies were also assessed for publication bias through a Funnel Plot.

## Results

### Evidence synthesis

#### Study selection and characteristics

**Fig. 1:**
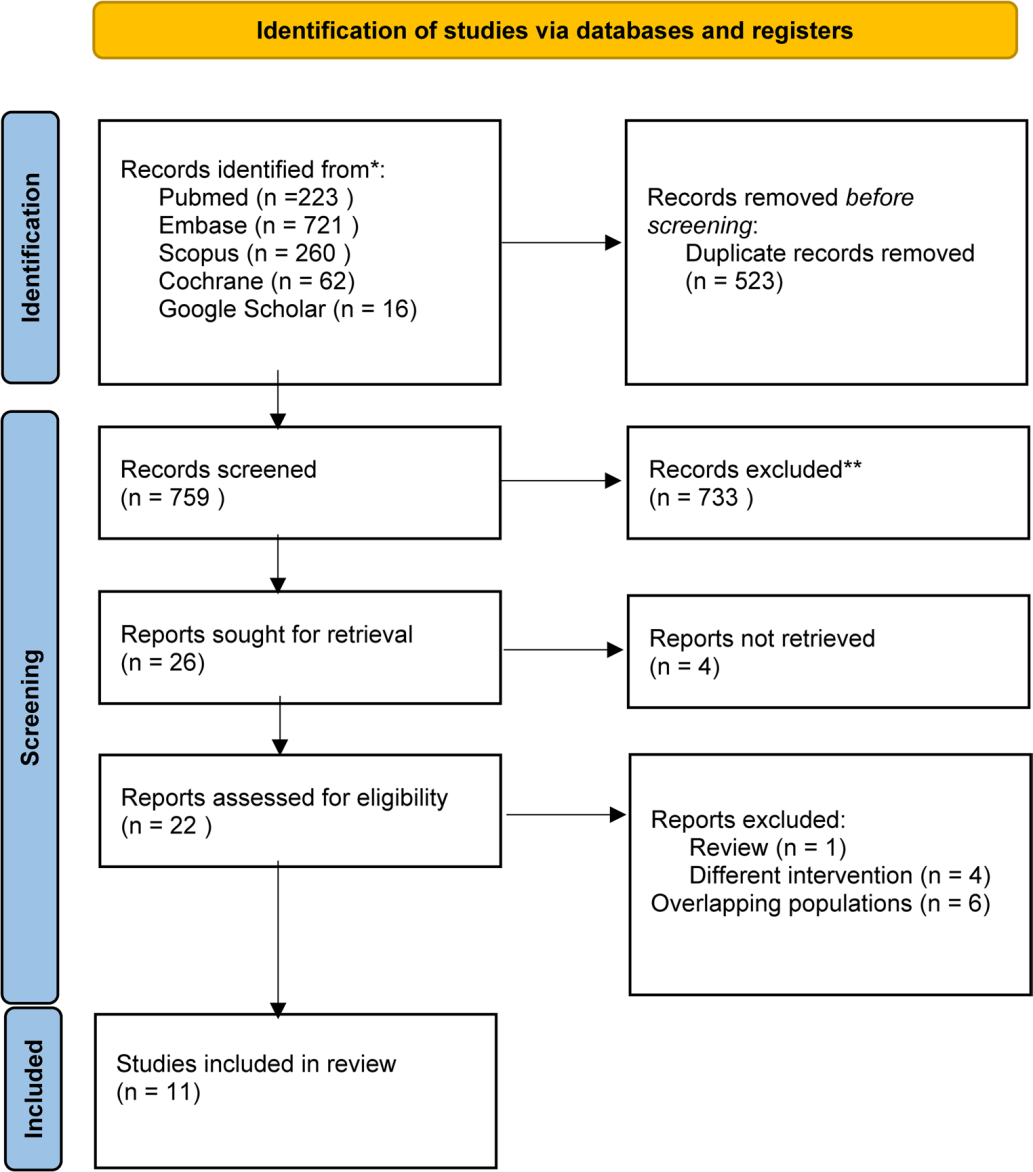
PRISMA screening flowchart

After searching the databases, 1282 articles were retrieved. After screening and removing duplicates, 11 articles were considered relevant and included in our analysis (Fig. 1). Table 1 provides an overview of the demographic data of the patients in all included studies.

Among the included studies, 5 were randomized controlled trials (RCTs), while the remaining 6 were not RCTs. Combining the data from these articles, 764 patients were analyzed in the CC group and 748 patients in the TRT group.

### Meta-analysis

**Table 1:** Baseline characteristics of the included studies of clomiphene versus testosterone

^Author^	^Country^	^Study design^	^Patients^ ^CC/ TRT^	^Age, years CC/ TRT^	^Clomiphene dosing^	^Testosterone^ ^dosing^	^Type of TRT^	^Hypogonadism treatment criteria^
Dadhich, 2017 [10]	USA	Prospective cohort	23/52	37/ 51	n/a	n/a	27 received testosterone injections and 25 men received testosterone gel.	serum testosterone < 300 ng/dL associated with ≥ 3 hypogonadal symptoms verified on the ADAM questionnaire
Wheeler, 2017 [11]	USA	Retrospective cohort	188/175	38/51	25 mg daily	n/a	intramuscular, transdermal gel, and subcutaneous pellet	total testosterone level less than 300 ng/dl
McCullough ZA-304, 2015 [12]	USA	RCT	41/43	49/47	n/a	Androgel 1,62%	Gel	morning testosterone < 300 ng/dL
Kim ZA -305, 2015 [13]	USA	RCT	44/42	47/ 45	12, 5 mg daily 25 mg daily	Androgel 1,62%	Gel	morning testosterone < 300 ng/dL
Mazzola, 2015 [14]	FRANCE	Retrospective cohort	142/164	44 / 58	25 mg daily to 50 mg daily	n/a	transdermal gel	two early morning serum TT levels < 300ng/dl
Wiehle, 2015 [15]	USA	RCT	32/20	53/51	12, 5 mg daily 25 mg daily	Androgel 1,62%	Gel	two early morning serum TT levels < 300ng/dl
Lee, 2015 [16]	USA	Retrospective cohort	121/137	40/40	25–50 mg daily	n/a	transdermal gel	n/a
Ramasamy (clomiphene vs. Injections), 2014 [17]	USA	Retrospective cohort	31/31	42/41	25 mg daily	testosterone cypionate 100-200 mg once a week intramuscularly	Injections	serum testosterone < 300 ng/dL associated with ≥ 3 hypogonadal symptoms verified on the ADAM questionnaire
Ramasamy (clomiphene vs. gels), 2014 [17]	USA	Retrospective cohort	31/31	41/44	25 mg daily	Testim 1% or Androgel 1.62%, 2–4 pumps/day	Gel	serum testosterone < 300 ng/dL associated with ≥ 3 hypogonadal symptoms verified on the ADAM questionnaire
Kaminetsky, 2013 [18]	USA	RCT	8/8	46/46	25 mg daily	Androgel 1%	Gel	morning testosterone < 300 ng/dL
Jolene, 2011 [19]	USA	RCT	46/14	53/54	6,25 mg daily 12,5 mgdaily 25 mg/dia	Androgel 1%	Gel	morning testosterone ≤ 350 ng/dL.
Taylor, 2010 [20]	USA	Retrospective cohort	65/39	65/39	50 mg every other day	Androgel^®^ 1% or Testim^®^ 1%	Gel	serum total T < 300 ng/dL) or male infertility

*RCT* Randomized controlled trial, *CC* Clomiphene, *TT* Testosterone replacement therapy

### Testosterone levels

**Fig. 2:**
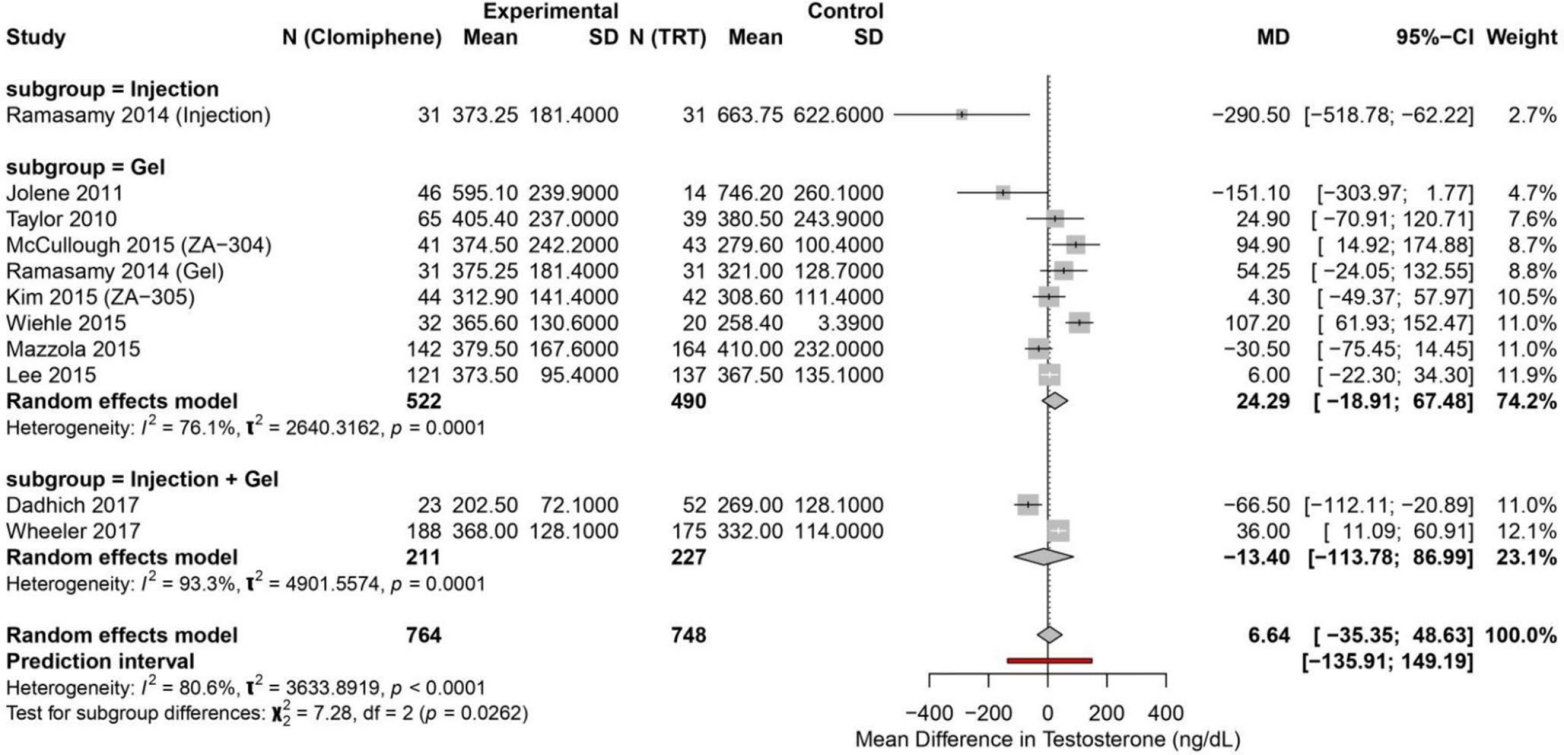
Forest plot (random-effects) - Clomiphene vs. TRT

**Fig. 3:**
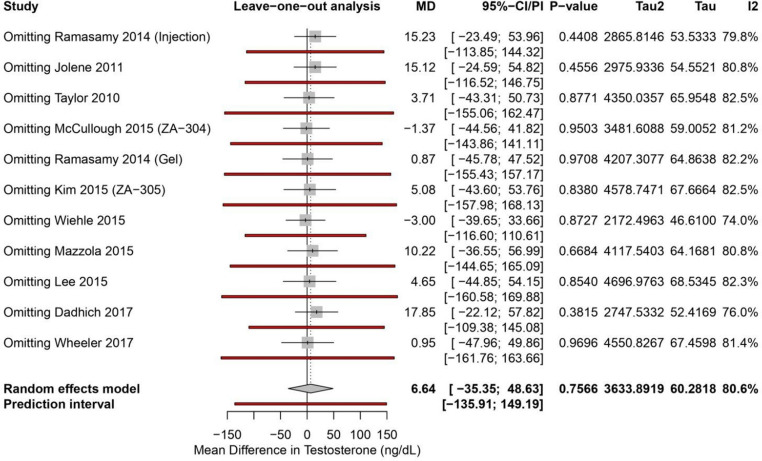
Leave-one-out sensitivity analysis for Testosterone levels

The random-effects forest plot (Fig. 2) comparing the change in serum testosterone levels between clomiphene and testosterone replacement therapy (TRT), stratified by TRT route (injection, gel, or mixed injection + gel) reveals a mean difference of 6.64 ng/dL (95% CI − 35.35 to 48.63) with an overall high heterogeneity (I² = 80.6%, τ² = 3633.89, Cochran’s Q *p* < 0.0001). Hence, a subgroup analysis, a Leave One Out analysis (Fig. 3) and a Baujat plot analysis were performed to address heterogeneity. Nevertheless, the magnitude of the pooled effect varies and heterogeneity remains high across iterations, reflecting limited precision of the pooled estimate. The Baujat plot (Supplementary Figure S1) suggests that Wiehle et al. [15] study was the most important study to increment heterogeneity, which was confirmed on the LOO, nevertheless I² heterogeneity still remained high (74%).

A funnel plot (Supplementary figure S2) was constructed to assess the distribution of study effect sizes (mean difference in testosterone levels, ng/dL) against their standard errors in order to visually evaluate potential small-study effects or publication bias. In the absence of bias, studies are expected to be symmetrically distributed around the pooled effect estimate. Visual inspection suggested a slight asymmetry toward the left side of the funnel. However, Egger’s linear regression test did not indicate statistically significant funnel plot asymmetry (*p* = 0.42).

### FSH levels

Serum FSH levels were reported in a limited number of studies with extreme heterogeneity (I² = 90.6%). Given the instability of the pooled estimate observed in sensitivity analyses, a quantitative meta-analytic synthesis was deemed inappropriate for this outcome. Individual study results are presented descriptively in Table 2. In general, clomiphene citrate was associated with numerically higher FSH levels compared with TRT, which is biologically consistent with the stimulatory effect of clomiphene on pituitary gonadotropin secretion versus the suppressive effect of exogenous testosterone. However, the magnitude and direction of the difference varied substantially across studies, precluding reliable conclusions.

**Table 2:** Individual study results for serum FSH levels (CC vs. TRT)

Author (Year)	*N* (CC/TRT)	FSH (CC), Mean ± SD	FSH (TRT), Mean ± SD	Mean Difference (IC95%)
Kaminetsky et al. (2013) [18]	8 / 8	1,55 ± 0,78	2,10 ± 1,55	-0,55 (-1,75 a 0,65)
Kim et al. (2015)	44 / 42	6,00 ± 1,41	5,50 ± 0,71	0,50 (0,03 a 0,97)
Wiehle et al. (2015) [15]	31 / 13	10,50 ± 6,36	4,50 ± 2,12	6,00 (3,48 a 8,52)

*CI* Confidence interval, *CC* Clomiphene citrate, *TRT* Testosterone replacement therapy

### LH levels

Serum LH levels were assessed in a limited number of studies with extreme heterogeneity (I² = 95.6%). Given these findings, quantitative pooling was not considered methodologically appropriate, and individual study results are presented descriptively in Table 3. Leave-one-out analysis demonstrated that the omission of Kaminetsky et al. [18] completely resolved heterogeneity (I² = 0%) and shifted the pooled estimate substantially, confirming that the summary result was driven by a single study. The consistently higher LH levels observed with clomiphene are biologically plausible, reflecting stimulation of the hypothalamic–pituitary–testicular axis, whereas TRT suppresses gonadotropins through negative feedback.

**Table 3:** Individual Studys Results for serum LH levels (CC vs. TRT)

Author (Year)	*N* (CC/TRT)	LH (CC), Mean ± SD	LH (TRT), Mean ± SD	Mean Difference (95% CI)
Kaminetsky et al. (2013) [18]	8 / 8	1.95 ± 0.21	1.95 ± 0.21	0.00 (-0.21 to 0.21)
Kim et al. (2015, ZA-305)	44 / 42	5.00 ± 1.41	3.50 ± 0.71	1.50 (1.03 to 1.97)
Mazzola et al. (2015) [14]	142 / 164	5.10 ± 1.40	3.80 ± 3.39	1.30 (0.73 to 1.87)

### ADAM

**Fig. 4:**
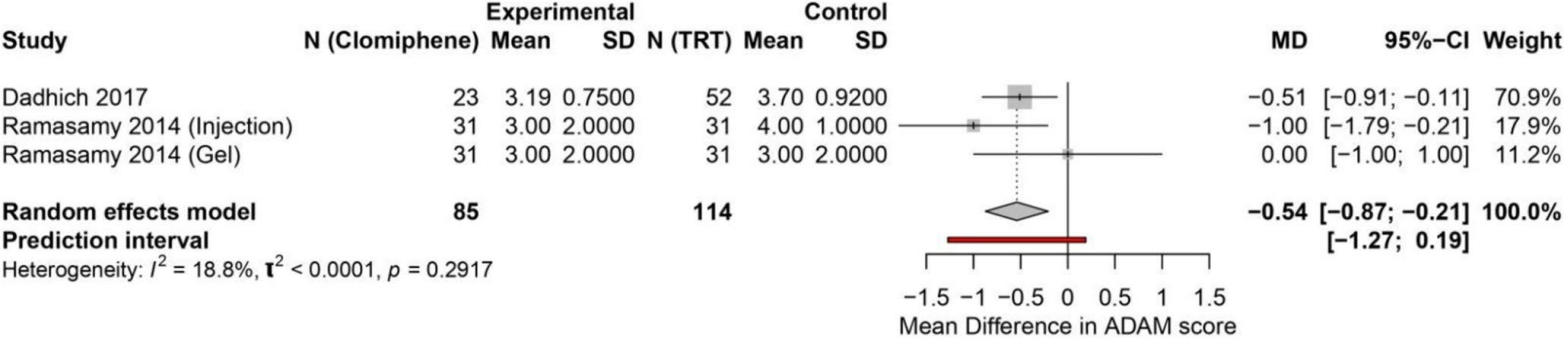
Forest plot of ADAM - libido domain

Three studies (199 patients) assessed ADAM questionnaire scores - libido domain (Fig. 4). Given the small number of studies, these results should be regarded as exploratory. In the random-effects meta-analysis, testosterone replacement therapy (TRT) was associated with a greater reduction in symptoms compared with clomiphene, with a mean difference of − 0.54 points (95% CI: −0.87 to − 0.21). Heterogeneity was low (I² = 18.8%). The effect favored TRT in studies using injectable formulations, whereas testosterone gel showed no significant difference compared with clomiphene.

### Quality assessment

**Fig. 5:**
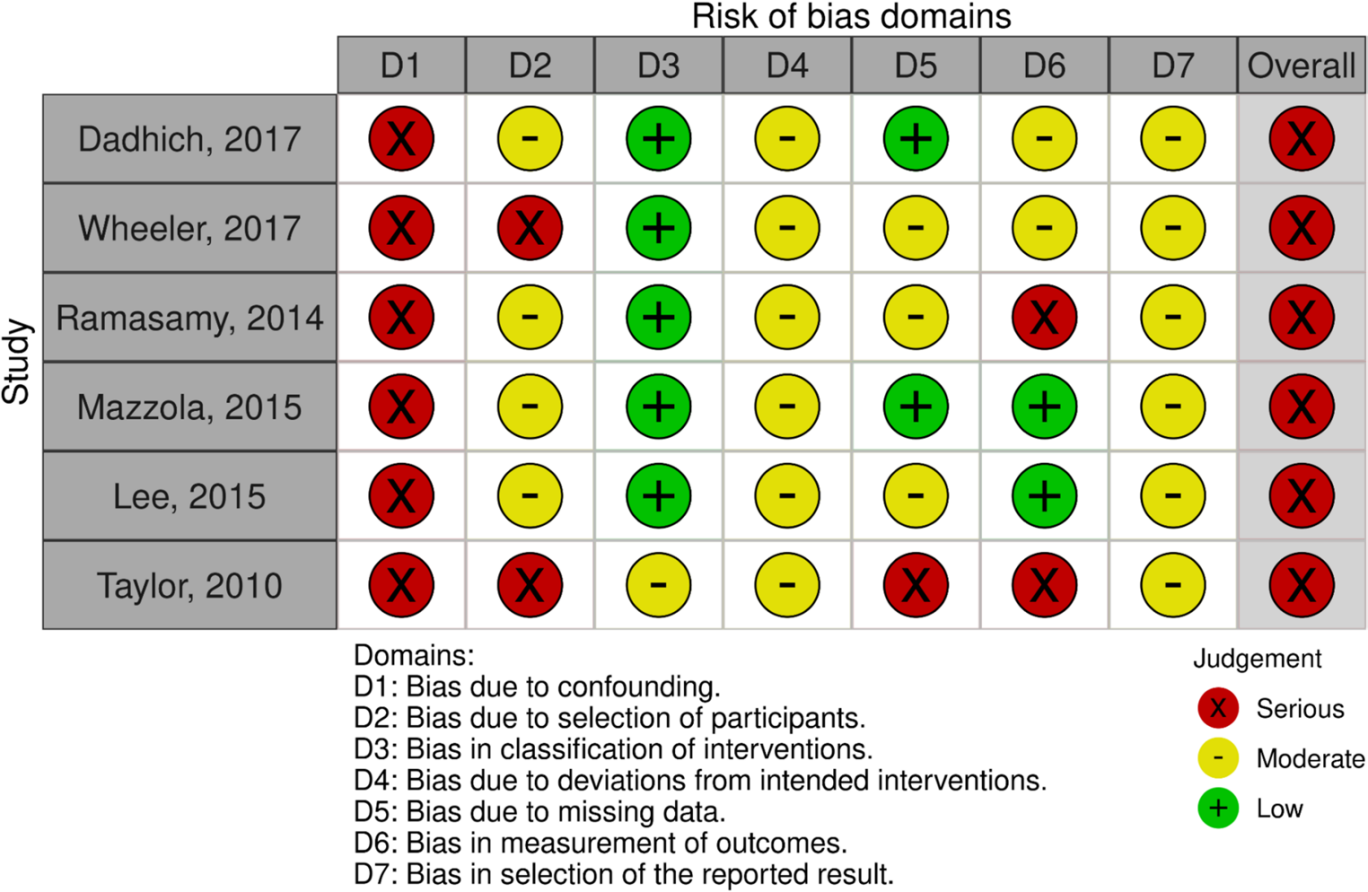
ROBINS - I tool

**Fig. 6:**
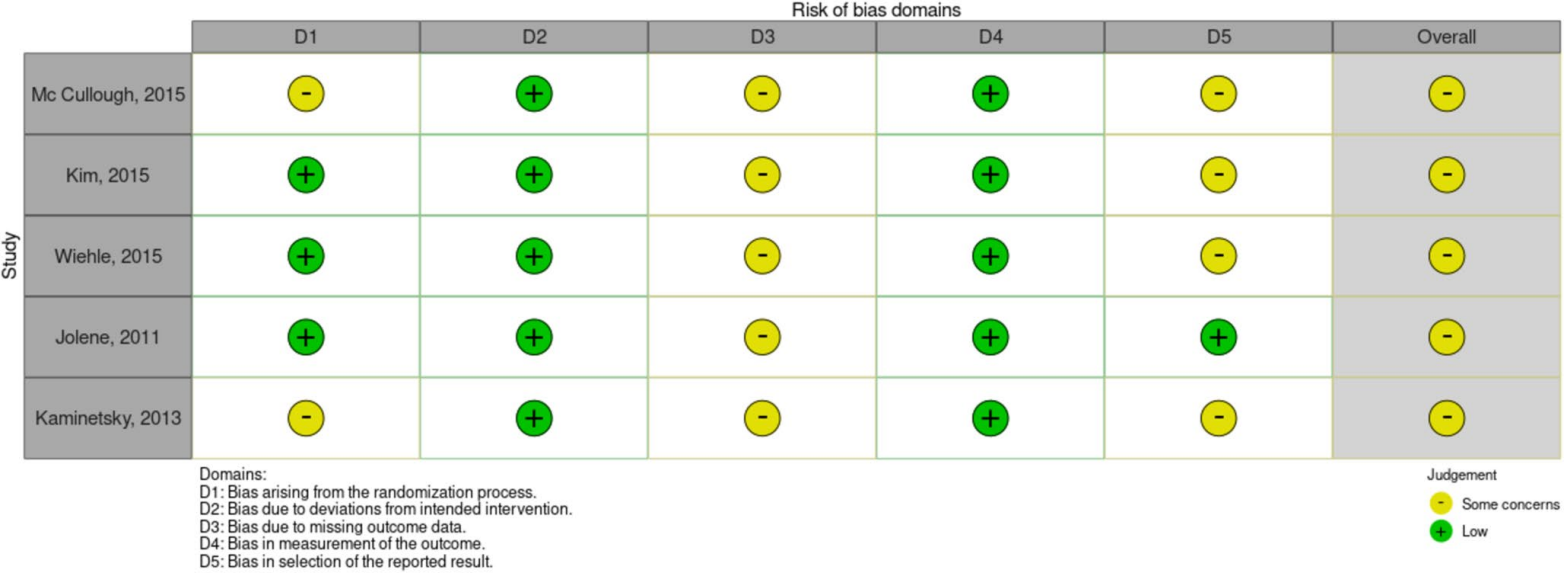
ROB- 2

The 6 non-randomized studies were assessed using the ROBINS-I tool (Fig. 5) and demonstrated a high risk of bias. On the other hand, the randomized studies were assessed using the RoB-2 tool (Fig. 6), with all 5 studies showing an overall judgement of some concerns.

### GRADE Assessment

Using the GRADE framework (Supplementary figure S3), the certainty of evidence was rated as very low for serum testosterone levels due to serious concerns regarding risk of bias (mixed study designs with moderate-to-high risk of bias), inconsistency (I2 = 81%, with opposite directions by route of administration), indirectness (different testosterone formulations grouped), and imprecision (wide 95% CI: -28.11 to 45.54 ng/dL). For libido assessed via the ADAM questionnaire, the certainty was rated as low, downgraded for risk of bias and imprecision (only 3 studies, *n* = 199). FSH and LH were rated as very low certainty due to extreme heterogeneity (I2 > 90%) and limited number of studies.

## Discussion

Previous studies demonstrate that clomiphene citrate is effective in increasing serum testosterone levels in men with hypogonadism, yielding results comparable to those achieved with testosterone replacement therapy [21]. In this systematic review and meta-analysis, we compared clomiphene citrate with testosterone replacement therapy (TRT) in the treatment of men with late-onset hypogonadism. Our pooled analysis did not demonstrate a statistically significant difference in serum testosterone levels between clomiphene and TRT across all studies combined; however, this finding must be interpreted with caution given the high between-study heterogeneity (I² = 80.6%) and the instability of the pooled estimate observed in sensitivity analyses [21, 22].

Serum total testosterone is the cornerstone biochemical parameter for diagnosing and monitoring treatment of male hypogonadism according to both the AUA⁵ and EAU¹ guidelines, which define deficiency thresholds and guide treatment titration based on this measure. The choice of serum testosterone as the primary outcome is therefore clinically justified. Nevertheless, biochemical normalization alone does not guarantee improvement in patient-reported symptoms.

The inclusion of both RCTs and non-randomized comparative studies was guided by the Cochrane Handbook for Systematic Reviews of Interventions, which recognizes that combining different study designs may be warranted when the available evidence from RCTs alone is insufficient to address the clinical question of interest. Given that only five RCTs were identified, restricting the analysis exclusively to RCTs would have precluded meaningful data pooling. Risk of bias was assessed separately for each study design using validated tools (RoB-2 for RCTs; ROBINS-I for non-randomized studies).

As expected, the single study assessing injectable testosterone formulations isolated was associated with significantly higher serum testosterone concentrations compared with clomiphene citrate. In contrast, studies comparing clomiphene with transdermal testosterone gel reported numerically similar serum testosterone levels, without statistically significant differences. However, despite biochemical equivalence, clomiphene was associated with lower libido compared with injectable testosterone formulations, as assessed by the sexual domain of the ADAM questionnaire.

This finding suggests that similar serum testosterone levels do not necessarily translate into equivalent clinical responses. Although TRT is expected to suppress both LH and FSH via negative feedback by hypothalamic–hypophysis–testicular axis [12, 14], LH suppression was consistently observed, when no significant difference in FSH levels was found between clomiphene citrate and transcutaneous TRT. This probably reflects the high heterogeneity and the distinct regulation of FSH, which is only partially androgen-dependent and strongly influenced by inhibin B and Sertoli cell function [11, 12, 15]. Variable androgen exposure with transcutaneous formulations and limited sample size may further explain this finding. Besides, the group suggests the possible variation due to differences in dihydrotestosterone levels, androgen peak exposure – with endogenous estradiol increase, resulting from physiological intratesticular and peripheral aromatization, tissue pharmacodynamics, or peripheral conversion [9, 16, 19, 21, 23].

It should be acknowledged that the quality of the included evidence was limited. The five RCTs were judged to have some concerns regarding risk of bias (RoB-2), while all six non-randomized studies carried serious/high risk of bias (ROBINS-I).These methodological limitations reduce confidence in the pooled estimates and preclude definitive conclusions regarding the comparative efficacy of clomiphene citrate and TRT.

An important consideration concerns sexual function. Our analyses demonstrated that libido-related outcomes were more favorable to TRT. In the three included studies that assessed this factor using the ADAM questionnaire, clomiphene was associated with significantly lower post-treatment libido scores compared to TRT. Consistent with our findings, clinical evidence suggests that while both clomiphene citrate and testosterone replacement therapy effectively increase serum testosterone levels, TRT tends to produce greater improvements in libido scores. In a comparative cohort, men treated with injectable testosterone reported significantly higher libido measures than those on clomiphene, and clomiphene treatment was associated with lower post-treatment libido sub-scores on validated symptom questionnaires [9, 22]. 

Regarding long-term treatment with clomiphene citrate, the study by Krzastek et al. demonstrated that it is a safe and effective option for men with hypogonadism. The authors reported a sustained and significant increase in serum testosterone levels over prolonged follow-up, accompanied by improvement in hypogonadal symptoms and high rates of patient satisfaction. Importantly, clomiphene citrate preserved endogenous gonadotropin production and spermatogenesis, distinguishing it from exogenous testosterone therapies. The treatment was well tolerated, with a low incidence of adverse effects and minimal discontinuation rates, suggesting that clomiphene citrate may warrant further investigation as a potential long-term option for men with hypogonadism who wish to preserve fertility [24]. According to the GRADE assessment, the certainty of evidence ranged from very low to low across all outcomes evaluated, primarily due to risk of bias, substantial heterogeneity, and the limited number of available studies. These findings indicate that the results of this study should be interpreted with caution, underscoring the need for additional, methodologically more robust studies to confirm the true magnitude and direction of the observed effects.

### Limitations

This review comprises the most comprehensive compilation of studies to date comparing clomiphene with testosterone replacement therapy (TRT). However, several limitations should be acknowledged. First, the included studies varied considerably in sample size, treatment dose, and follow-up duration. In addition, clomiphene and TRT differ substantially in their mechanisms of action and onset of therapeutic effect, with clomiphene typically requiring a longer period to achieve stable endogenous testosterone levels compared with the more rapid response observed with TRT. Therefore, differences in treatment duration and timing of outcome assessment across studies may have led to either underestimation or overestimation of the relative efficacy of clomiphene citrate, and contributed to interstudy heterogeneity. Second, the analysis of sexual function was based on only three studies with relatively small cohorts. Third, heterogeneity - particularly in testosterone-related outcomes—was substantial, underscoring the need for standardized protocols and more homogeneous patient populations. Moreover, although preservation of fertility is cited as one of the main theoretical advantages of clomiphene over TRT, none of the included studies directly assessed fertility outcomes such as semen parameters, conception rates, or time to pregnancy. This constitutes a critical gap, and future studies should prioritize the inclusion of fertility endpoints. Finally, the available data was insufficient to allow a quantitative assessment of hematological and prostate-related complications, limiting conclusions regarding the comparative safety profiles of clomiphene and TRT. Furthermore, although the title specifies late-onset hypogonadism (LOH), several included studies enrolled patients based solely on a serum total testosterone threshold of < 300 ng/dL, without requiring age-specific or symptom-based criteria consistent with LOH as defined by the EAU. This population heterogeneity — encompassing functional, mixed, and true age-related hypogonadism — limits the specificity of the conclusions and should be considered when applying these findings to a clinical LOH population. An additional limitation is that some included studies evaluated enclomiphene instead of racemic clomiphene citrate. Although related compounds, they are not identical, and combining both interventions may have introduced indirectness into the pooled analysis. Because of the limited number of available studies, no sensitivity analysis restricted to clomiphene citrate was performed. Finally, clomiphene citrate and TRT differ substantially in their onset of therapeutic effect. TRT increases serum testosterone within days, whereas clomiphene — acting indirectly through HPG axis stimulation — typically requires several weeks to months to achieve stable testosterone levels. Because the included studies did not consistently report treatment duration prior to outcome measurement, relative efficacy comparisons may reflect different phases of the therapeutic response curve, introducing a systematic bias that cannot be fully addressed in the current analysis.

## Conclusion

In this systematic review and meta-analysis, no statistically significant difference in serum testosterone levels was observed between clomiphene citrate and testosterone gel formulations in men with late-onset hypogonadism; however, this should not be interpreted as evidence of non-inferiority, as no formal non-inferiority analysis was conducted. Furthermore, clomiphene was associated with lower libido scores compared with TRT. Given the moderate-to-high risk of bias across included studies, the high heterogeneity of most outcomes, and the paucity of studies, there is clearly a need for well-designed randomized clinical trials with standardized clinical outcomes — including fertility, sexual function, and quality of life — to clarify the comparative efficacy of these treatments in LOH.

## Supplementary Information

Supplementary Material 1.

## Data Availability

The data analyzed in this study were obtained from previously published articles identified through systematic searches of PubMed, Embase, Scopus, The Cochrane Library, and Google Scholar. All data supporting the findings of this study are included within the article. No new primary data were generated.
