# Supplementary material for: Clomiphene citrate versus testosterone replacement therapy in male hypogonadism: a systematic review of literature and meta-analysis
Source: Eur J Clin Pharmacol. 2026 Jul 11;82(8):204. doi: 10.1007/s00228-026-04134-3 (PMC13356072; doi:10.1007/s00228-026-04134-3)
Supplement: Supplementary file 1 — Supplementary Material 1. [file 228_2026_4134_MOESM1_ESM.docx]

**/Supplementary Material**


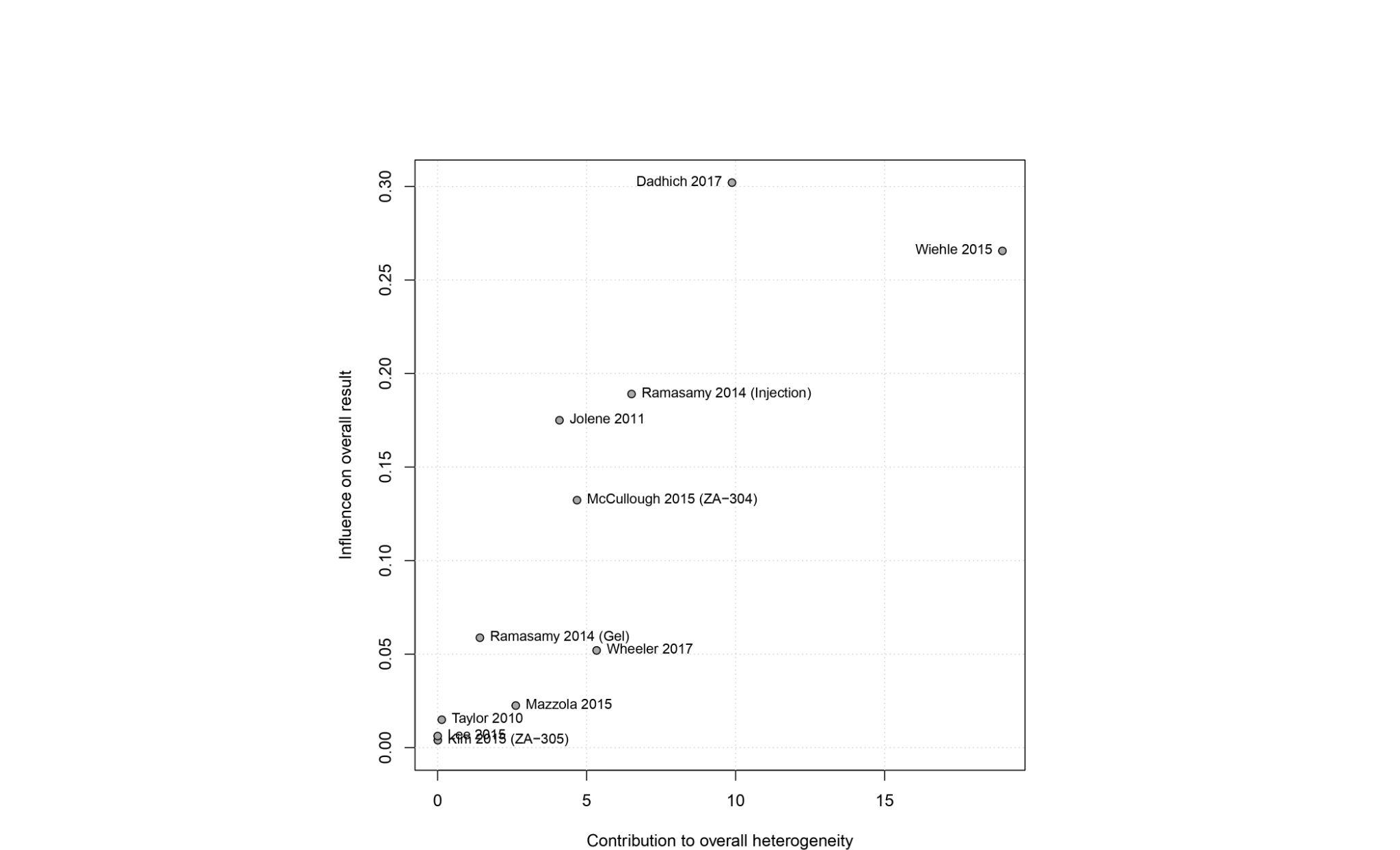


**Supllementary Figure S1.** Baujat plot (influence vs heterogeneity contribution)


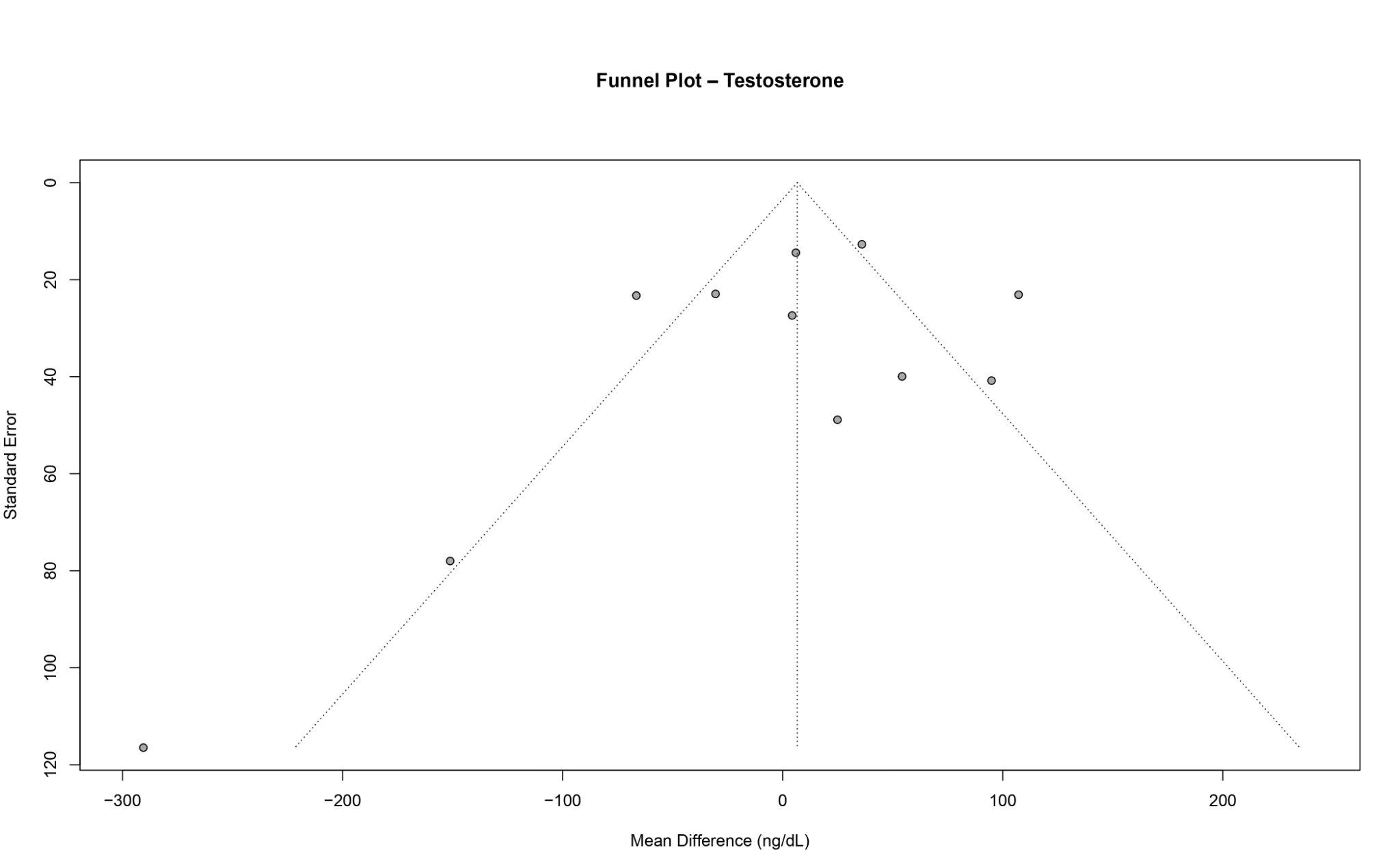


**Supllementary Figure S2.** Funnel plot Testosterone

**
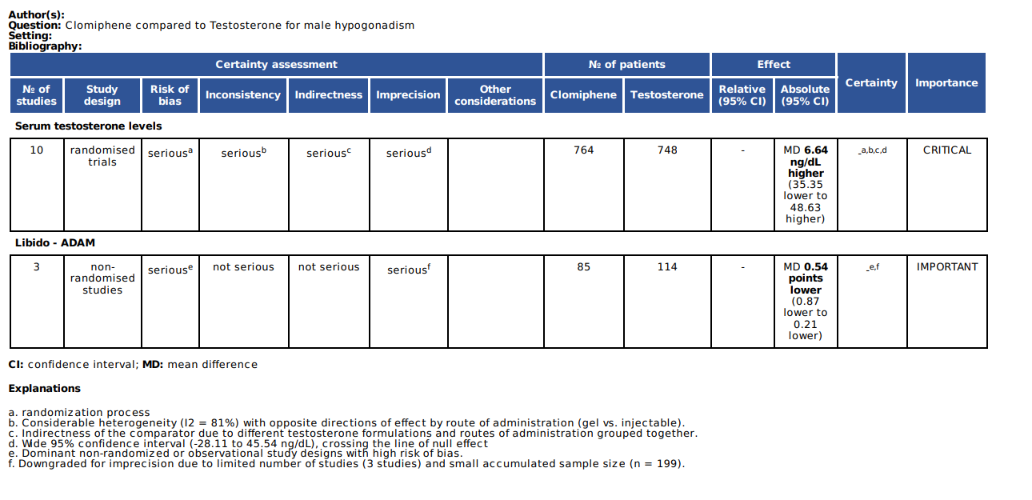
**

**Supllementary S3. GRADE Assessment**
